# BCLXL PROTAC degrader DT2216 targets secondary plasma cell leukemia addicted to BCLXL for survival

**DOI:** 10.3389/fonc.2023.1196005

**Published:** 2023-07-17

**Authors:** Ophélie Champion, Alana Soler, Sophie Maïga, Céline Bellanger, Catherine Pellat-Deceunynck, Alexis Talbot, Cyrille Touzeau, Martine Amiot, Patricia Gomez-Bougie

**Affiliations:** ^1^ Nantes Université, Inserm, CNRS, Université d’Angers, Centre de Recherche en Cancérologie et Immunologie Intégrée Nantes Angers (CRCI2NA), Nantes, France; ^2^ Département d’hématologie, Centre Hospitalier Universitaire (CHU) de Nantes, Nantes, France; ^3^ Département d’Immuno-hématologie, Hopital Saint-Louis, Assistance Publique Hôpitaux de Paris, Paris, France

**Keywords:** plasma cell leukemia, BCL2 family, BCLXL degrader, PROTAC, DT2216, multiple myeloma

## Abstract

Secondary plasma cell leukemia (sPCL) is a rare form of aggressive plasma cell malignancy arising mostly at end-stage refractory multiple myeloma and consequently presenting limited therapeutic options. We analyzed 13 sPCL for their sensitivity to BH3 mimetics targeting either BCL2 (venetoclax) or BCLXL (A1155463) and showed that 3 sPCL were efficiently killed by venetoclax and 3 sPCL by A1155463. Accordingly, BH3 profiling of 2 sPCL sensitive to BCLXL inhibition confirmed their high BCLXL primed profile. While targeting BCLXL using BH3 mimetics induces platelets on-target drug toxicity, the recent development of DT2216, a clinical-stage BCLXL proteolysis targeting chimera PROTAC compound, provides an alternative strategy to target BCLXL. Indeed, DT2216 specifically degrades BCLXL via VHL E3 ligase, without inducing thrombocytopenia. We demonstrated in human myeloma cell lines and sPCL that sensitivity to DT2216 strongly correlated with the sensitivity to A1155463. Interestingly, we showed that low doses of DT2216 (nM range) were sufficient to specifically degrade BCLXL after 48 hours of treatment, consistent with VHL expression, in all cell lines but irrespectively to DT2216 sensitivity. In myeloma cells, DT2216 induced apoptotic cell death and triggered BAX and BAK activation. In conclusion, our study demonstrated that patients with sPCL addicted to BCLXL, a small but a very challenging group, could potentially receive therapeutic benefit from DT2216. Clinical trials of DT2216 in this subset of sPCL patients are warranted.

## 1 Introduction

Apoptosis is mainly controlled by BCL2 protein family members and occurred when BH3-only pro-apoptotic proteins are sufficient to restrain the anti-apoptotic counterparts (MCL1, BCL2 and BCLXL) and activate BAX and BAK effector proteins (1, 2). The mechanism by which BAX and BAK convert from inactive monomers to active oligomers is a multistep process involving both changes in conformation and cellular localization (3). Once BAX/BAK oligomerize and form pores, they induce the permeabilization of the outer mitochondrial membrane, leading to cytochrome c release and apoptosis. While evasion to apoptosis is a hallmark of cancer cells, hematologic cancer cells are often characterized by a high apoptotic priming, mostly due to the upregulation of BCL2 anti-apoptotic members, following selection pressures during malignant transformation (4). This feature highlights the interest of BH3 mimetics to therapeutically exploit the addiction to BCL2 anti-apoptotic proteins (5). Multiple myeloma is a very heterogeneous pathology, which is also manifested by its diverse addiction to the three main anti-apoptotic molecules such as BCL2, BCLXL and MCL1 for survival (6–8). The addiction on these anti-apoptotic proteins relies on their capacity to sequester and restrain their pro-apoptotic counterparts, mechanism that is favored by the overexpression of these pro-survival members observed in MM (7, 9). Of note, MM dependence on MCL1 and BCLXL for survival increases at relapse (8). To date, the only clinically available BH3 mimetic is venetoclax, which targets BCL2. However, targeting BCLXL and MCL1 leads to adverse events, such as thrombocytopenia and cardiotoxicity, respectively (10–12). Currently, proteolysis targeting chimera (Protac) technology allowed the development of a safe first-in-class drug DT2216, targeting BCLXL via the VHL E3 ligase (13). Indeed, DT2216 inhibits several xenograft tumors without inducing significant thrombocytopenia (13, 14) and it is currently in Phase 1 trials in relapsed/refractory malignancies (NCT04886622).

Secondary plasma cell leukemia (sPCL) is a rare but highly aggressive form of plasma cell dyscrasias, occurring in patients at a late and advanced stage of aggressive multiple myeloma. Since these patients are mostly refractory to conventional and novel therapies, they consequently have a dismal outcome and limited therapeutic options (15). Recently, the clinical activity of venetoclax has been reported in sPCL (16). Because BCLXL Protac degrader allows targeting BCLXL without inducing significant thrombocytopenia, we hypothesized that targeting BCLXL in sPCL could have a potential interest. In the current study, we demonstrated that a subgroup of sPCL exhibited a BCLXL primed profile and therefore could be selectively killed by DT2216. We then addressed the mechanism of action of DT2216 in myeloma cells with a particular attention on the activation of BAX and BAK.

## 2 Materials and methods

### 2.1 Primary plasma cells and human myeloma cell lines

After informed consent, blood samples from multiple myeloma patients were collected at the University Hospital of Nantes Department of Hematology (MYRACLE study; NTC03807128) (17). For the present study, inclusion criteria were adult man/woman, diagnosis of relapsed multiple myeloma, presence of circulating plasma cells (≥5%). Blood from one patient (sPCL1) was collected at the department of Immuno-hematology of Saint-Louis Hospital (Paris). Cells from this patient were immortalized in our laboratory after being cultured in RPMI1640 with 5% FCS and 3ng/ml recombinant IL-6 and gave rise to NAN12 cell line.

Human myeloma cell lines (HMCLs, n=10) were extensively characterized as previously described (18). The XG5, XG7, MDN, NAN10 and NAN12 HMCLs were derived in our laboratory. The KMS12PE and KMM1 HMCLs were kindly provided by Dr. Otsuki (Kawasaki Medical School, Kurashiki, Japan); KARPAS-620 (K620), by Dr. Karpas (Cambridge Clinical School, Cambridge UK); ANBL6 by Dr. Jelinek (Rochester,USA) and MM1S, by Dr. S. Rosen (Northwestern University, Chicago, USA).

### 2.2 Reagents and antibodies

Venetoclax (ABT-199) and A1155463 were purchased from Selleck Chemicals GmbH, DT2216 from Clinisciences. The following antibodies were used: BCL2 (Dako, M0887), BCLXL (2764S), BAX (2772), BAK (12105), PUMA (12450) and VHL (68547) from Cell Signaling. BCLXL (sc-271121), MCL1 (sc-12756), BIK (sc-10770) from Santa Cruz. ACTIN (MAB1501) and BIM (Ab17003) from Millipore.

### 2.3 Cell death assays

Mononuclear cells (MNC) were isolated from blood samples by Ficoll-Hypaque density gradient centrifugation and immediately cultured in RPMI-1640 media with 5% fetal calf serum. Plasma cells were identified using CD138 staining (anti-CD138-PE, Beckman Coulter). MNC were incubated 24h with BCL2, BCLXL BH3 mimetics or DT2216 and an untreated condition was included as a control. After CD138 staining, cell death response was measured by the loss of CD138 expression, as previously described (8). Specific cell death was expressed as the percentage relative to the untreated control condition.

Cell death in HMCL was assessed using AnnexinV-FITC staining. Fluorescence acquisition and analysis were performed using a FACsCanto (Becton Dickinson) and FlowJo software.

### 2.4 Intracellular BH3 profiling and cytochrome *c* release

BH3 profiling was performed using the BIM BH3 derived peptide (0.1 μM), HRK BH3 derived peptide (10 μM) and the inert recombinant PUMA BH3-only peptide (PUMA2A) as negative control, as previously described (19). Briefly, cells were permeabilized with 0.004% Digitonin and exposed to peptides for 45 min at 27°C before fixation with 8% formaldehyde at room temperature for 15 min. After addition of neutralizing buffer (Tris 0.41 mol/L glycine pH 9.1) for 5 min, cells are stained with anti-cytochrome *c*–Alexa 647 (BLE612310, Ozyme) 1:40 in 0.1%Saponin/1%BSA/PBS overnight at 4°C. Loss of cytochrome *c* was analyzed by a flow cytometry. The quantification of cytochrome *c* loss induced by each peptide was analyzed by gating the cytochrome *c* negative population. For cytochrome *c* release, cells were treated with DT2216 for 15h, then permeabilized with 0.004% Digitonin and processed as in BH3 profiling.

### 2.5 Isolation of CD138 positive cells and Gene expression analysis

Plasma cells were isolated from the MNC fraction using CD138 immunomagnetic beads, MS columns and MACS™ Separator (Miltenyl Biotech) following manufacture’s protocol. Briefly, cells were magnetically labeled with the CD138 immunomagnetic beads during 15 minutes at 4°C. The MS column was placed on the MACS™ Separator and pre-washed with MACS buffer. Then, cell suspension was loaded into the column and the flow-through fraction, containing the CD138 negative cells, was discarded. After 3 washes with MACS buffer, the column was removed from the separator. The retained cells were eluted as the CD138 + fraction.

Total RNA was obtained from purified CD138+ plasma cells using RNeasy mini kit (Qiagen). The mRNA expression of BCL2 family members was performed by 3’digital gene expression (DGE) RNA-sequencing protocol as previously described (20).

### 2.6 Immunoblotting and Immunoprecipitation

Cells were lysed for 40 minutes on ice in 0.5% NP40 containing buffer for immunoblotting or lysed in 1% Digitonin containing buffer for co-immunoprecipitation assays. Lysates were centrifugated at 12,000 X g for 30 min and supernatants were collected. Western blotting and immunoprecipitation reactions were performed as previously described (21). Protein expression levels were quantified using ImageLab and Image J software.

### 2.7 BAX and BAK activation

For the detection of active forms of BAX and BAK, 5x10^5^ cells were treated with DT2216 or not. After incubation, cells were fixed and permeabilized using the FOXP3 transcription factor staining buffer set (Thermo Fischer Scientific, 00-5523-00) following the manufacturer’s recommendations. Cells were incubated with the following antibodies: BAX (clone 6A7, Santa Cruz, sc-23959), BAK (clone G317-2, BD Biosciences) and a mouse Ig G1 isotypic control (Miltenyl, 130-106-545) for 30 min. After washing, cells were incubated with the Alexa fluor 647 antibodies for 30 min, washed once in PBS and resuspended in PBS-1% formaldehyde. The flow cytometry acquisition and analysis were performed on a FacsCanto (Becton Dickinson) and FlowJo software, respectively.

### 2.8 Statistical analysis

Correlation was assessed by the Spearman correlation method, (*r*) and *p* values are indicated.

## 3 Results

### 3.1 Analysis of the sensitivity to BCL2 and BCLXL BH3 mimetics in primary sPCL

**Figure 1:**
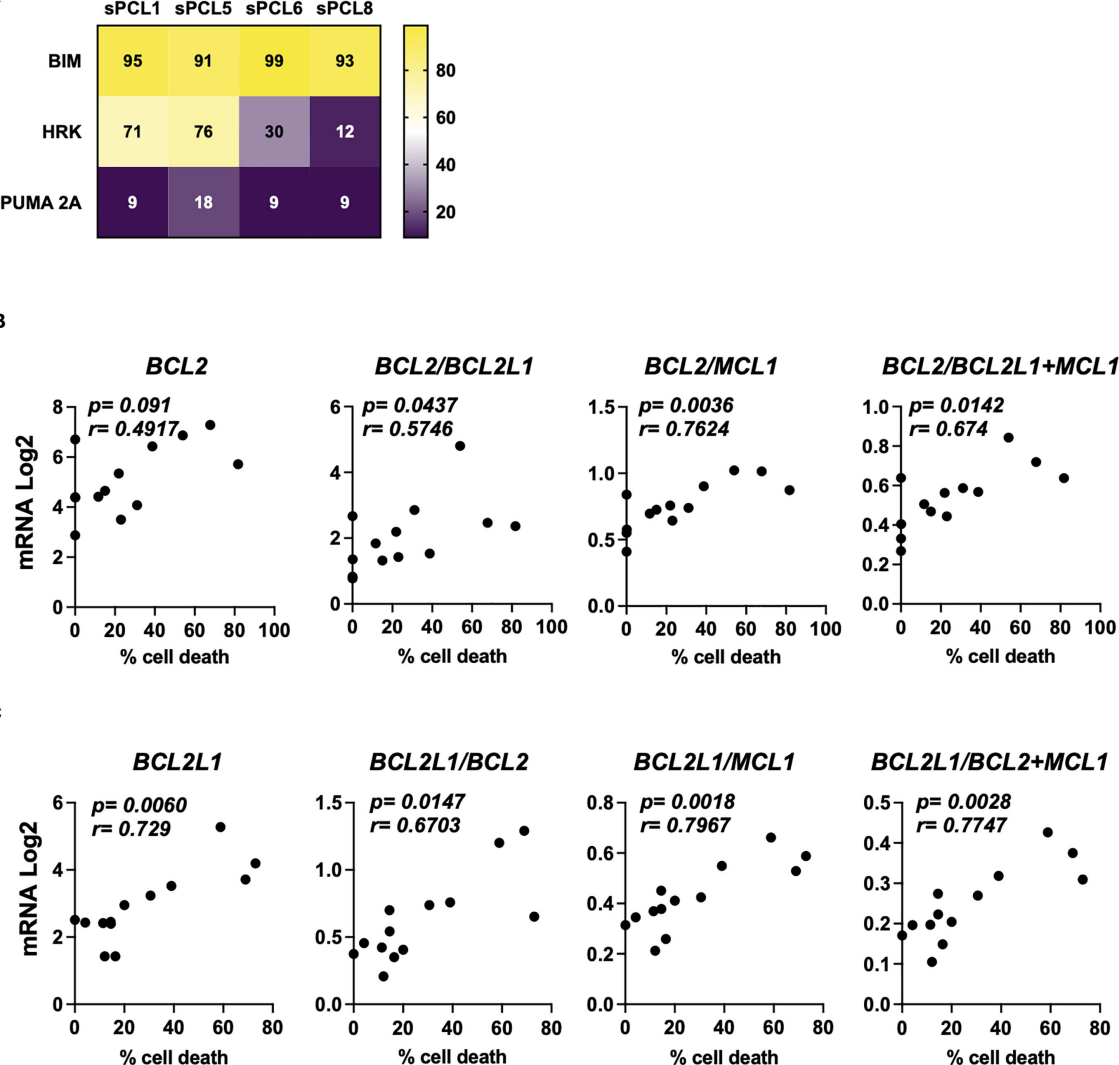
Characterization of BCL2 or BCLXL primed profile of sPCL. **(A)** BH3 profiling was performed exposing CD138 positive cells isolated from patient samples to BH3-derived peptides, values corresponded to the percentage of cytochrome *c* released and measured by flow cytometry. Bim peptide measures global priming, HRK peptide measures BCLXL dependence and PUMA 2A is an inert peptide. **(B)** Analysis of *BCL2*, *BCL2/BCL2L1*, *BCL2/MCL1* and *BCL2/BCL2L1+MCL1* mRNA seq expression according to venetoclax (300nM) cell death response in primary cells from sPCL (n=13). *BCL2L1* is the BCLXL coding gene. **(C)** Analysis of *BCL2L1, BCL2L1/BCL2, BCL2L1/MCL1, BCL2L1/BCL2+MCL1* mRNA seq expression according to A1155463 (300nM) cell death response in primary cells from sPCL (n=13). Correlation was assessed by Spearman test, *p* and *r* values are indicated.

A cohort of 13 consecutive sPCL was analyzed for the sensitivity to BH3 mimetics targeting either BCL2 (ABT-199/venetoclax) or BCLXL (A1155463) (**Table 1**). The main molecular characteristics of sPCL were defined on the basis of DGE RNA sequencing profile and are summarized in **Table 1**. sPCL 4, 6 and 8 were only sensitive to venetoclax, sPCL 1, 5 and 11 were sensitive to A1155463 and the other 7 sPCLs were resistant to both BH3 mimetics (**Table 1**). Analysis of cell death in sPCL samples upon BH3 mimetics treatment is illustrated for sPCL1, sensitive to A1155463 and for the non-sensitive sPCL3 sample (**Supplementary Figure 1**). Additionally, sensitivity to A1155463 was paired with BH3 profiling using BIM-BH3 derived peptide, to evaluate the global priming and HRK-BH3 derived peptide, specific for BCLXL, in four sPCL samples; sPCL1 and sPCL5 were sensitive to A1155463 and sPCL6 and sPCL8 sensitive to venetoclax. This analysis showed 91 to 99% cytochrome c release after exposure to BIM-derived peptide in the four analyzed samples, reflecting their high capacity to undergo mitochondrial apoptosis, according to their sensitivity to the respective BH3 mimetic. However, only cells from sPCL1 and sPCL5 permeabilized their mitochondria upon the HRK-BH3 peptide (71 and 76% cytochrome c release, respectively), confirming the BCLXL primed profile of these samples (**Figure 1A**). In contrast, cells from sPCL6 and sPCL8 released 12 and 30% of cytochrome c upon exposure to HRK-BH3 peptide, in agreement with their resistance to A1155463 (**Figure 1A**).

**Table 1:** *Ex vivo* sensitivity of sPCL cells to BH3 mimetics.

sPCL characteristics				Cell death %	
N°	Age/sex	Molecular group	Del17p	ABT199 (300nM)	A1155463 (300nM)
1	57/F	MF	+	0	59
2	66/F	MS	ND	31	16
3	64/M	HD	ND	22	4
4	69/F	CD2	ND	82	11
5	67M	MF	+	0	69
6	56/F	MS	+	68	20
7	72/F	MF	–	12	14
8	69/F	CD1	–	54	12
9	61/M	CD1	+	0	15
10	84/M	LB	ND	15	39
11	69/F	CD2	+	38	73
12	48/M	LB	+	0	30
13	36/M	LB	ND	0	0

Blood mononuclear cells were treated with ABT199 (Venetoclax) or A1155463, both at 300 nM for 24 hours. The percentage of cell death was determined by the loss of CD138 staining. sPCL, secondary plasma cell leukemia; F, female; M, male; ND, not determined. Molecular groups were established as previously described (22).

We further analyzed the mRNA BCL2 family expression according to BH3 mimetics response. We found that *BCL2* mRNA levels did not correlated with venetoclax sensitivity in contrast to *BCL2/BCL2L1* (p=0.0437), *BCL2/BCL2L1+MCL1* (p=0.0142) and *BCL2/MCL1* (p=0.0036) mRNA ratios, the latter ratio correlated the best with venetoclax response, (**Figure 1B**). Of note, the sensitivity to A1155463 significantly correlated with *BCL2L1* (coding for BCLXL) mRNA levels (p=0.006), though the ratio of *BCL2L1/MCL1* (p=0.0018) mRNA appeared the most potent marker associated with A1155463 sensitivity (**Figure 1C**). Nevertheless, the MCL1 mRNA levels itself did not correlate with the sensitivity of any of both BH3 mimetics tested. (**Supplementary Figure 2**).

### 3.2 BCLXL Protac DT2216 efficiently degrades BCLXL in all myeloma cells

**Figure 2:**
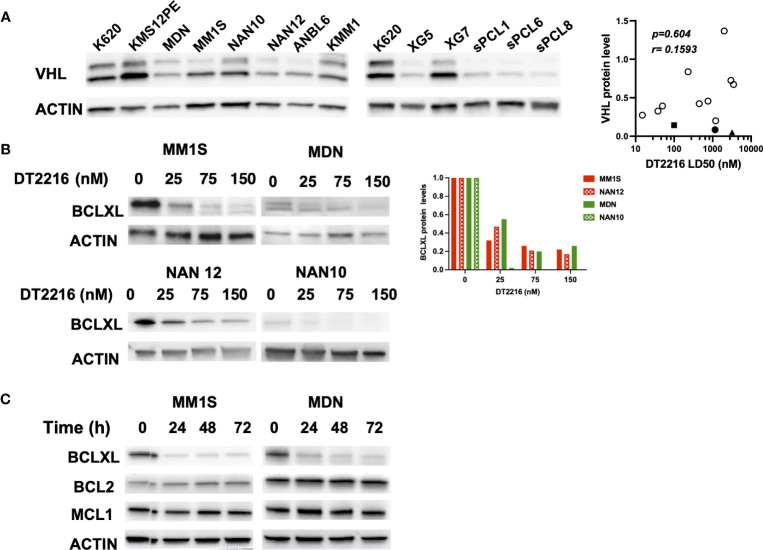
DT2216 selectively degrades BCLXL in myeloma cells. **(A)** Immunoblot of VHL expression in HMCLs (n=10) and CD138 (+) sPCL (n=3). KARPAS 620 (K620) cell lysate was included as an internal control. Results are representative of 2 independent experiments (left panel). VHL relative protein levels were quantified, normalized to actin and plotted against DT2216 LD50 values. Correlation was assessed by Spearman test, *p* and *r* values are indicated (right panel). **(B)** HMCLs were treated for 48h by DT2216 as indicated. Cell lysates were analyzed by western blot for BCLXL and Actin expression. BCLXL protein levels were quantified using Actin as a loading control. Results are representative of 2 independent experiments. **(C)** HMCLs were treated by DT2216 (150nM) for the indicated times. Cell lysates were analyzed by western blot. Results are representative of 2 independent experiments.

Since the sPCLs sensitive to A1155463 were venetoclax resistant, targeting BCLXL by the specific BCLXL Protac degrader DT2216 could be of interest for this particular sPCL subgroup. To study the specificity and the ability of DT2216 to degrade BCLXL, we took advantage of myeloma cell lines (HMCLs) and in particular of NAN12 cell line, which has been immortalized in presence of IL-6 from the sPCL1. Because DT2216 relies on Von Hippel-Lindau (VHL) E3 ligase to achieve BCLXL protein degradation, we first analyzed VHL expression in 10 HMCLs and CD138+ cells from 3 sPCL (1, 6 and 8). Due to alternative codon initiation, VHL protein is expressed as two isoforms of 18 and 24 kDa (23), which were detected in all HMCL tested (n=10), as well as in sPCL cells (**Figure 2A**). However, the levels of the VHL E3 ligase did not correlate with DT2216 sensitivity (r=0.1593, p=0.604) (**Figure 2A**, **Supplementary Table 1**), suggesting that cell death induced by DT2216 was not influenced by the level of expression of VHL protein. We next analyzed the degradation of BCLXL in 4 different cell lines treated with various doses of DT2216. After 48hrs of treatment, 20 to 50% of BCLXL was already degraded at 25nM DT2216, reaching a major degradation of at least 80% of BCLXL observed at 150nM in all HMCLs tested (**Figure 2B**). Finally, we confirmed that DT2216 selectively degraded BCLXL, even after 72 hours at the dose of 150nM, without modifying the levels of MCL1 or BCL2 (**Figure 2C**).

### 3.3 DT2216 induces apoptosis in highly expressing BCLXL cells through BAX and BAK activation

**Figure 3:**
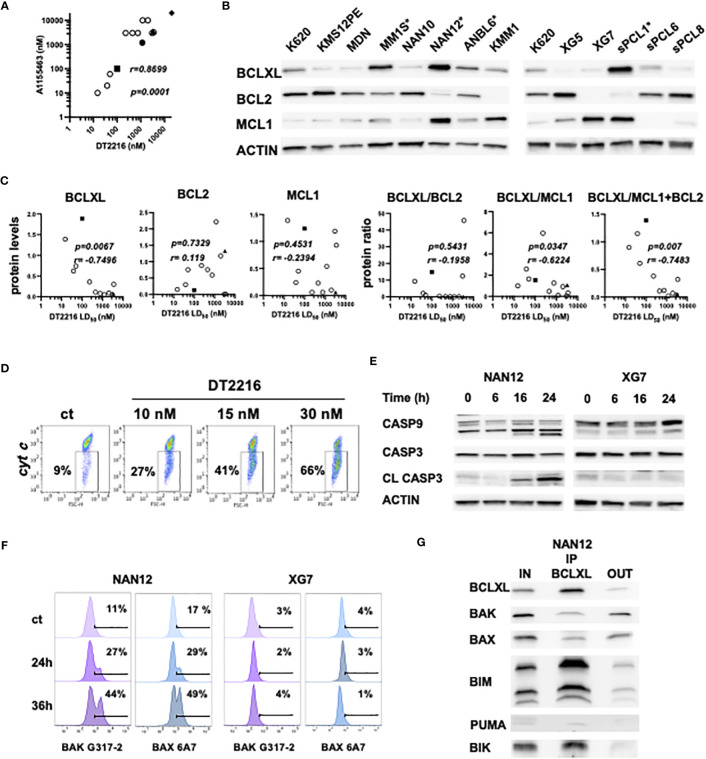
DT2216 induces apoptosis in myeloma cells through BAK and BAX activation. **(A)** Sensitivity to DT2216 and A1155463 correlates in myeloma cells. DT2216 LD50 values were plotted versus A1155463 LD50 values in 10 HMCLs (empty circles) and 4 sPCLs (sPCL1=square; sPCL6=circle; sPCL8=triangle and sPCL13=diamond). Correlation was assessed by Spearman test, *p* and *r* values are indicated. **(B)** BCLXL, BCL2 and MCL1 protein levels were analyzed by western blotting. KARPAS 620 (K620) cell lysate was included as an internal control. * DT2216 sensitive cells. **(C)** BCLXL, BCL2 and MCL1 protein levels were quantified and normalized to actin. DT2216 LD50 values were analyzed in function to relative protein levels and the protein ratio of BCLXL/BCL2, BCLXL/MCL1 and BCLXL/MCL2+BCL2. Correlation was assessed by Spearman test, *p* and *r* values are indicated. **(D)** NAN12 cells were treated by DT2216 for 15h, followed by an intracellular staining using an anti-cytochrome *c* mAb and analyzed by flow cytometry. The percentage of cells that released cytochrome *c* upon DT2216 is indicated. Results are representative of 2 independent experiments. **(E, F)** NAN12 and XG7 were treated for the indicated times with 15nM and 150nM DT2216, respectively. Cells were collected and analyzed by **(E)** western blotting or **(F)** stained with anti-BAK (G-317-2) and anti-BAX (6A7) mAb antibodies directed against their active forms. Results are representative of at least 3 independent experiments. **(G)** BCLXL Immunoprecipitation was performed in cell lysate from NAN12 cell line. Unbound proteins (OUT) were quantified and compared to total lysates (IN). Results are representative of 2 independent experiments.

We further compared the sensitivity to DT2216 and A1155463 in HMCLs (n=10) and primary cells from sPCLs (1, 6, 8 and 13). We found a strong and significant correlation between the sensitivity to these two compounds (r=0.8699, p=0.0001, Spearman test). Three cell lines (ANBL6, MM1S and NAN12) and 1 sPCL (sPCL1) were highly sensitive to DT2216 with LD_50_ values ranging from 15 to 100 nM (**Figure 3A**, **Supplementary Table 1**). Because 150nM of DT2216 almost completely degraded BCLXL (at least 80%) in all myeloma cells studied, only cells that were significantly killed at this dose or less were considered as BCLXL dependent. Moreover, DT2216 sensitive cells were resistant to venetoclax (**Supplementary Table 1**) and therefore dependent on BCLXL. One cell line (KARPAS 620) had a particular sensitivity profile characterized by an intermediate sensitivity to DT2216 (LD_50 _=_ _231 nM) and a high sensitivity to venetoclax (LD_50 = _5 nM). This result indicates a co-dependence on BCLXL as well as BCL2, which may rely on its high BCL2 protein level (**Figure 3B**).

We also analyzed the expression of the main BCL2 anti-apoptotic proteins in relation to DT2216 response. DT2216 sensitive myeloma cells expressed the highest levels of BCLXL and moderate levels of either BCL2, MCL1 or both. In opposition, resistant cells were characterized by a lower BCLXL and higher BCL2 and/or MCL1 protein expression (**Figure 3B**). We further demonstrated that DT2216 sensitivity strongly correlated with BCLXL protein expression (r= -0.7496, p=0.0067) as well as the ratio of BCLXL/MCL1+BCL2 protein (r=-0.7483, p=0.007) (**Figure 3C**). Although to a lesser extent, the protein ratio of BCLXL/MCL1 also correlated with induction of cell death by DT2216 (r=-0.6224, p=0.0347), while no correlation was found with the ratio of BCLXL/BCL2. At the same time, neither BCL2 nor MCL1 showed any correlation with DT2216 cell death induction (**Figure 3C**). To go deeper into the mechanism of cell death induction by DT2216, we investigated its impact on the mitochondrial apoptotic pathway. Indeed, we demonstrated cytochrome *c* release (**Figure 3D**), caspase-9 and consequently caspase-3 activation under DT2216 treatment in sensitive NAN12 cells. In contrast, no caspase activation was observed in the resistant XG7 cell line (**Figure 3E**). Of note, using antibodies against the conformational active forms of BAK and BAX, we demonstrated that upon DT2216 treatment both effectors were readily activated from 24hrs (27% BAK and 29% BAX) in NAN12 cells. Activation that clearly progressed at 36 hrs, since around 50% of cells displayed both activated forms. As expected, no activation of BAK or BAX was observed in the resistant XG7 cells (**Figure 3F**).

Finally, we investigated the nature of BCLXL complexes in the sensitive NAN12 cell line by co-immunoprecipitation assays. Our results revealed that BCLXL was complexed with both BH3-only (BIK, BIM) and effector proteins (BAX, BAK) (**Figure 3G**). Quantification of pro-apoptotic proteins before and after BCLXL immunoprecipitation indicated that 86% of BIK, 77% of BIM and around 50% of BAX and BAK effectors expressed in NAN12 were sequestered by BCLXL. Thus, these findings supported the activation of BAX and BAK effectors in DT2216 cell death induction, according to the unified model of the BCL2 members interaction (1).

## 4 Discussion

The present study demonstrates that the novel platelet-sparing BCLXL protac degrader, DT2216, selectively degrades BCLXL in all myeloma cells tested but only kills cells highly primed for BCLXL. The specificity of DT2216 is determined by the recruitment of BCLXL to VHL E3 ligase (13), which we found expressed in sPCL and HMCL cells analyzed. Nevertheless, the protein level of VHL E3 ligase did not correlate with DT2216 cell death induction, as already reported in T-cell acute lymphoblastic leukemia cell lines (24). In contrast, BCLXL protein levels strongly correlated with DT2216 cell death, suggesting that the level of BCLXL protein expression dictates the sensitivity to DT2216 in tumor plasma cells. Accordingly, it has been demonstrated that T cell lymphoma cells expressing high BCLXL levels were highly sensitive to DT2216 (14). The same study found that high MCL1 expressing cells were resistant to DT2216, similarly to our results observed in XG7 MM cell line. These observations suggest that MCL1 could act as a brake for cell death induced by DT2216.

It is worth noting that low concentrations of DT2216 (nM range) were needed to induce myeloma cell death compared to the high doses used in solid tumor cells (25). Indeed, similar to our findings, low doses of DT2216 were found to kill BCLXL dependent T cell lymphomas *in vitro* and *in vivo* as well as T−cell acute lymphoblastic leukemia cells (13, 24). Whether high DT2216 sensitivity could be extended to other hematological cancers remains undefined.

Despite the low number of patient samples included in this study, the identification of a subgroup of sPCL addicted to BCLXL, but not to BCL2, for survival fully illustrated the importance of targeting BCLXL for these end-stage refractory patients with a dismal outcome (26). Additionally, our preliminary analysis of bone marrow samples from relapse/refractory MM patients showed that of the 22 samples analyzed, only 2 were sensitive to DT2216 (cell death ≥ 50% at 150nM), suggesting that a small group of relapse/refractory MM patients are dependent on BCLXL and could potentially receive therapeutic benefit from DT2216. However, larger number of samples are needed to confirm this finding.

We could hypothesize that in DT2216 sensitive cells, the degradation of BCLXL could trigger the release of its bound counterparts, namely BH3-only proteins and BAX/BAK effectors. Thus, freed BH3-only proteins may in turn activate BAX and BAK (Mode 1). Not mutually exclusive, the release of BAX and BAK effectors from BCLXL may also lead to their activation (Mode 2), as already described (27, 28). The concomitant activation of BAX and BAK observed under DT2216 suggests the formation of BAX/BAK heterocomplexes at the mitochondria, as previously described in myeloma cells (20). Of note, we demonstrated that a major pool of BIK was bound to BCLXL in NAN12 sensitive cells, this BH3-only protein, long described as a sensitizer, is now considered as direct activator of BAX and BAK (29, 30).

Of samples that were sensitive to venetoclax, sPCL 4 and sPCL 8 belonged to CD-2 and CD-1 molecular groups, which are characterized by an over-expression of CCND1 and the presence of t(11;14) (22). Indeed, several studies demonstrated the potential clinical use of venetoclax in MM patients harboring t(11;14) (31–33). Furthermore, the likelihood of a clinical response was associated with high BCL2/MCL1 and BCL2/BCL2L1 mRNA ratios (31, 32). Accordingly, we confirmed the role of MCL1 as a resistance factor for venetoclax response in our cohort of sPCL. Interestingly, our results propose that in sPCL, MCL1 could also play a role in resistance to BCLXL targeting. Of note, the gene coding for MCL1 is located in 1q21, region frequently amplified in MM (34).

Because previous studies demonstrated that the functional evaluation of venetoclax sensitivity predicts clinical response (33), we can expect that the functional evaluation of DT2216 sensitivity could also predict the clinical response of sPCL to DT2216. From our results, we conclude that this functional *ex vivo* assays, rapidly and easily analyzed by flow cytometry after 48 hours DT2216 treatment, will be the mandatory partner of personalized approach and represents the missing link between omic data of an individual and clinical application for each patient. Besides the interest of DT2216 to target cancer cells that rely on BCLXL for survival, it was recently shown that DT2216 could improve cancer immunotherapy by eliminating regulatory T cells (Tregs) (35). Thus, the study of Kolb et al. broadens the potential use of BCLXL protact degrader, which is already under clinical trials (NCT04886622).

Finally, even if sPCL addicted to BCLXL remains a small yet a very challenging group of patients, DT2216 appears an additional weapon that merits a clinical evaluation.

## Data availability statement

The original contributions presented in the study are included in the article/**Supplementary Materials**, further inquiries can be directed to the corresponding author/s.

## Ethics statement

The studies involving human participants were reviewed and approved by University Hospital of Nantes (MYRACLE study; NTC03807128). The patients/participants provided their written informed consent to participate in this study. Written informed consent was obtained from the individual(s) for the publication of any potentially identifiable images or data included in this article.

## Author contributions

OC, AS, SM and CB performed experiments and participated in the design of the study. AT provided patient samples. CP-D participated in the design of the study and reviewed the paper, CT provided patient samples and reviewed the paper, PG-B and MA performed experiments, designed the study and wrote the paper. All authors contributed to the article and approved the submitted version.
