# Supplementary material for: BCLXL PROTAC degrader DT2216 targets secondary plasma cell leukemia addicted to BCLXL for survival
Source: Front Oncol. 2023 Jul 17;13:1196005. doi: 10.3389/fonc.2023.1196005 (PMC10393035; doi:10.3389/fonc.2023.1196005)
Supplement: Supplementary file 1 [file DataSheet_1.docx]

**Supplementary Figure 1**.

**BH3 mimetics *ex vivo* testing of sPCL samples**

Mononuclear cells obtained from sPCL1 and sPCL3 were cultured in the presence of ABT-199 (300nM), A1155463 (300nM) or untreated (control condition) for 24h. Cells were then stained with anti-CD138-PE mAb. Tumor plasma cell death was assessed by the loss of CD138 expression. The percentage of CD138 positive cells in each condition after 24h of culture is indicated. Specific cell death for each BH3 mimetic was calculated relative to control (ct) and is indicated in brackets.
