# Supplementary material for: BCLXL PROTAC degrader DT2216 targets secondary plasma cell leukemia addicted to BCLXL for survival
Source: Front Oncol. 2023 Jul 17;13:1196005. doi: 10.3389/fonc.2023.1196005 (PMC10393035; doi:10.3389/fonc.2023.1196005)
Supplement: Supplementary file 2 [file DataSheet_2.docx]

**Supplementary Figure 2**.

**MCL1 mRNA levels did not correlated with either Venetoclax or A1155463 cell death response in sPCL samples.**

Analysis of *MCL1* mRNA expression according to A) venetoclax (300 nM) and B) A1155463 (300nM) cell death response. Correlation was assessed by Spearman test, *p* and *r* values are indicated.
