# Supplementary material for: BCLXL PROTAC degrader DT2216 targets secondary plasma cell leukemia addicted to BCLXL for survival
Source: Front Oncol. 2023 Jul 17;13:1196005. doi: 10.3389/fonc.2023.1196005 (PMC10393035; doi:10.3389/fonc.2023.1196005)
Supplement: Supplementary file 3 [file DataSheet_3.docx]

**Reference Supplementary Table 1**

Moreaux J, Klein B, Bataille R, Descamps G, Maïga S, Hose D, Goldschmidt H, Jauch A, Rème T, Jourdan M, Amiot M, Pellat-Deceunynck C. A high-risk signature for patients with multiple myeloma established from the molecular classification of human myeloma cell lines. Hematologica (2011) 96(4):574-82. doi: 10.3324/haematol.2010.033456
