# Supplementary material for: BCLXL PROTAC degrader DT2216 targets secondary plasma cell leukemia addicted to BCLXL for survival
Source: Front Oncol. 2023 Jul 17;13:1196005. doi: 10.3389/fonc.2023.1196005 (PMC10393035; doi:10.3389/fonc.2023.1196005)
Supplement: Supplementary file 4 [file Table_1.docx]

| **HMCL/sPCL** | **Translocation** | **Target Genes** | **LD_50_ (nM)** | | |
| --- | --- | --- | --- | --- | --- |
|  |  |  | **ABT199** | **A1155463** | **DT2216** |
| KARPAS 620 | t(11 ;14) | *CCND1* | 5 | 3000 | 231 |
| KMS12PE | t(11 ;14) | *CCND1* | 15 | > 10000 | 2000 |
| MDN | t(11 ;14) | *CCND1* | 3 | 3000 | 450 |
| NAN10 | t(11 ;14) | *CCND1* | 350 | > 3000 | 800 |
| XG5 | t(11 ;14) | *CCND1* | 5 | > 10000 | 1200 |
| KMM1 | t(6 ;14) | *CCND3* | 5000 | > 4000 | > 4000 |
| MM1S | t(14 ;16) | *c-Maf* | 4000 | 20 | 38 |
| ANBL6 | t(14 ;16) | *c-Maf* | 5500 | 60 | 50 |
| XG7 | t(4 ;14) | *MMSET* | 5000 | > 3000 | > 3000 |
| NAN12 | t(14 ;20) | *MafB* | 2000 | 10 | 15 |
| sPCL1 | t(14 ;20) | *MafB* | n.d. | 100 | 100 |
| sPCL6 | t(4 ;14) | *MMSET* | n.d. | 1200 | 1160 |
| sPCL8 | t(11 ;14) | *CCND1* | n.d. | 3500 | 3200 |
| sPCL13 | n.d. | n.d. | n.d. | >20000 | >20000 |

**Supplementary Table 1**

For human myeloma cell lines (HMCL), cell death was determined after 24 hours of ABT199 (Venetoclax) or A1155463 treatment or after 48 hours of DT2216 treatment. Cell death was assessed by flow cytometry after Annexin V staining. Values corresponded to the mean of at least 3 independent experiments. For sPCL samples, cell death was assessed by the loss of CD138 expression, as presented in Supplementary Figure 1. Target genes were previously defined (36). n.d. (not determined).
